# The evolution of performing a kidney biopsy: a single center experience comparing native and transplant kidney biopsies performed by interventional radiologists and nephrologists

**DOI:** 10.1186/s12882-022-02860-1

**Published:** 2022-06-25

**Authors:** Daria Emelianova, Marios Prikis, Christopher S. Morris, Pamela C. Gibson, Richard Solomon, Geoffrey Scriver, Zachary T. Smith, Anant Bhave, Joseph Shields, Michael DeSarno, Abhishek Kumar

**Affiliations:** 1 Department of Radiology, Robert Larner MD College of Medicine, Burlington, VT USA; 2 Division of Nephrology and Transplantation, Department of Medicine, Robert Larner MD College of Medicine, Burlington, VT USA; 3 Department of Pathology, Robert Larner MD College of Medicine, Burlington, VT USA; 4 Department of Medicine, Robert Larner MD College of Medicine, Burlington, VT USA; 5 Department of Medical Biostatistics, Robert Larner MD College of Medicine, Burlington, VT USA; 6 Yale School of Medicine, Section of Nephrology, New Haven, CT USA

**Keywords:** Interventional Radiology, Native Kidney, Nephrology, Renal Biopsy, Transplant Kidney

## Abstract

**Background:**

Kidney biopsy is the most vital tool guiding a nephrologist in diagnosis and treatment of kidney disease. Over the last few years, we have seen an increasing number of kidney biopsies being performed by interventional radiologists. The goal of our study was to compare the adequacy and complication rates between kidney biopsies performed by interventional radiology versus nephrology.

**Methods:**

We performed a single center retrospective analysis of a total of all kidney biopsies performed at our Institution between 2015 and 2021. All biopsies were performed using real-time ultrasound. Patients were monitored for four hours post biopsy and repeat ultrasound or hemoglobin checks were done if clinically indicated. The entire cohort was divided into two groups (Interventional radiology (IR) vs nephrology) based on who performed the biopsy. Baseline characteristics, comorbidities, blood counts, blood pressure, adequacy of the biopsy specimen and complication rates were recorded. Multivariable logistic regression was used to compare complication rates (microscopic hematuria, gross hematuria and need for blood transfusion combined) between these two groups, controlling for covariates of interest. ANCOVA (analysis of variance, controlling for covariates) was used to compare differences in biopsy adequacy (number of glomeruli per biopsy procedure) between the groups.

**Results:**

446 kidney biopsies were performed in the study period (229 native and 147 transplant kidney biopsies) of which 324 were performed by IR and 122 by nephrologist. There was a significantly greater number of core samples obtained by IR (mean = 3.59, std.dev. = 1.49) compared to nephrology (mean = 2.47, std.dev = 0.79), *p* < 0.0001. IR used 18-gauge biopsy needles while nephrologist exclusively used 16-gauge needles. IR used moderate sedation (95.99%) or general anesthesia (1.85%) for the procedures more often than nephrology, which used them only in 0.82% and 0.82% of cases respectively (*p* < 0.0001). Trainees (residents or fellows) participated in the biopsy procedures more often in nephrology compared to IR (97.4% versus 69.04%, *p* < 0.0001). The most frequent complication identified was microscopic hematuria which occurred in 6.8% of biopsies. For native biopsies only, there was no significant difference in likelihood of complication between groups, after adjustment for covariates of interest (OR = 1.01, C.I. = (0.42, 2.41), *p* = 0.99). For native biopsies only, there was no significant difference in mean number of glomeruli obtained per biopsy procedure between groups, after adjustment for covariates of interest (F(1,251) = 0.40, *p* = 0.53).

**Conclusion:**

Our results suggest that there is no significant difference in the adequacy or complication rates between kidney biopsies performed by IR or nephrology. This conclusion may indicate that kidney biopsies can be performed safely with adequate results either by IR or nephrologists depending on each institution’s resources and expertise.

## Introduction

Biopsy of a native or transplant kidney is the most vital tool guiding a nephrologist to diagnosis and treatment of kidney disease [1–3]. Kidney biopsy remains the gold standard in diagnosing kidney disease and has been a core part of clinical nephrology practice and training for decades. The technique used to obtain a kidney biopsy has evolved over the last seventy years, from performing the procedure without imaging guidance to using real-time ultrasound guidance, leading in improvement of the quality of the specimen, patient safety, and reducing complications [4–6]. Prompt diagnosis and treatment often hinges on an expedient, safe, and efficacious acquisition of an adequate kidney core needle biopsy specimen. However, questions remain about which clinical service is best prepared to meet these demands at a given institution. Until recently, both general and transplant nephrologists have been performing the majority of their patients’ kidney biopsies, with only few complicated cases done by interventional radiologists (IR). Over the last few years, at many institutions, an increasing number of native and transplant kidney biopsies are being performed by IR [7]. The reasons behind this paradigm shift may have been several, including time constraints, safety concerns, liability, credentialing requirements, expediency, and institution regulations.

Beginning three years ago, at our institution, our IR service began to perform all medical native and transplant kidney core needle biopsies. This shift in practice was mainly due to logistical and staffing reason. IR already had the resources in place to perform several procedures while the division of nephrology suffered from staffing shortages. Thus, the executive decision was made to request all kidney biopsies to be performed by IR. Following this change, we have conducted a retrospective observational study of all native and transplant kidney biopsies performed at the University of Vermont Medical Center, comparing safety and efficacy of kidney biopsies performed by nephrologists and radiologists.

## Methods

Biopsies performed by nephrology were done under ultrasound guidance at a radiology suite with the help of an ultrasound technologist for imaging. An immediate post biopsy ultrasound was performed by the technologist to assess for any evidence of bleeding. Subsequently, a sandbag was placed over. The biopsy site and the patients were monitored for four hours with frequent blood pressure monitoring. Thereafter, the patients were discharged home if they could urinate and there was no macroscopic hematuria. Biopsies done by IR were also done under real time ultrasound guidance in the IR suite. Similar to biopsies performed by nephrology, an ultrasound was performed immediately post-biopsy to assess for hematoma. The patients were also observed for four hours and then discharged home if able to urinate without macroscopic hematuria. No serum hemoglobin or repeat ultrasound was done again before discharge in both cases unless clinically indicated. Kidney biopsies were mostly done as a same day outpatient procedure.

A retrospective analysis of the electronic medical records from the University of Vermont Medical Center was performed. The University of Vermont Research Protections Office and Committees on Human Subjects for the University of Vermont and the University of Vermont Medical Center and Institutional Review Board of University of Vermont Medical Center determined that this project was exempt from review by the Institutional Review Board and provided a waiver of informed consent (study number 00001051). Data on kidney biopsies during a consecutive six-year period, performed between 2015 and 2021 at our institution was retrospectively collected and analyzed. Comparisons of native and transplant kidney biopsies were made between nephrologists and IR. Our primary outcome variables were biopsy adequacy (number of glomeruli available for light microscopy), average number of core needle biopsy samples, and presence of arteries. Secondary outcomes were incidence of complications like microscopic hematuria lasting more than 24 h, gross hematuria lasting more than 24 h, need for hospitalization or blood transfusion, need for surgical or other intervention, urinary tract infection or biopsy site infection, pain lasting more than 12 h, need for pain medications, inadvertent puncture of liver, pancreas, or spleen, and arteriovenous fistula formation. Baseline characteristics of the patients are presented in Table 1.

**Table 1:** Baseline patient characteristics between interventional radiology and nephrology

	**Interventional Radiology (*n* = 324)**	**Nephrology (*n* = 122)**	***p*-value**
**Age (years)**	54.74 (16.72)	56.43 (19.47)	0.26
**Systolic Blood Pressure (mmHg)**	133.82 (18.26)	130.28 (16.54)	0.07
**Diastolic Blood Pressure (mmHg)**	75.38 (13.69)	72.33 (14.13)	0.09
**INR**	1.05 (0.16)	0.99 (0.10)	**0.0002**
**PT (sec)**	12.16 (1.93)	11.47 (2.92)	** < 0.0001**
**BUN (mg/dl)**	42.44 (26.09)	39.52 (27.07)	0.07
**Platelet Count (x1000µl)**	240.90 (88.22)	246.53 (83.59)	0.29
**GFR (ml/min)**	34.68 (25.50)	44.98 (32.80)	**0.01**
**Creatinine (mg/dl)**	2.91 (2.64)	3.16 (5.64)	**0.04**
**Hemoglobin (g/dl)**	10.70 (2.40)	12.01 (2.37)	** < 0.0001**
**Hematocrit (%)**	32.23 (6.92)	35.49 (6.78)	** < 0.0001**
**Gender**^a^			0.86
Male	189 (58.33)	70 (57.38)	
Female	135 (41.67)	52 (42.62)	
**Race**^a^			0.06
White	287 (88.85)	111 (90.98)	
Black	18 (5.57)	1 (0.82)	
Other	18 (5.57)	10 (8.20)	
**Days Prior to Procedure Labs Collected**^a^			0.17
Less than or equal to 2 weeks	268 (83.23)	108 (88.52)	
Greater than 2 weeks	54 (16.77)	14 (11.48)	
**Type of Sedation**^a^			** < 0.0001**
No Sedation (Local Only)	7 (2.16)	120 (98.36)	
Moderate Sedation (Versed, fentanyl, etc.)	311 (95.99)	1 (0.82)	
General Anesthesia	6 (1.85)	1 (0.82)	
**Use of Opioids Prior to Procedure**^a^			0.21
No	270 (83.59)	107 (88.43)	
Yes	53 (16.41)	14 (11.57)	
**Fellow Involved in Procedure**^a^			** < 0.0001**
No	100 (30.96)	3 (2.46)	
Yes	223 (69.04)	119 (97.54)	
**Biopsy Performed Using CT Guidance**^a^			**0.02**
No	310 (95.98)	122 (100.00)	
Yes	13 (4.02)	0 (0.00)	
**Kidney**^a^			** < 0.0001**
Right	164 (50.77)	16 (13.11)	
Left	159 (49.23)	106 (86.89)	

Legend: *SBP* Systolic blood pressure, *DBP* Diastolic blood pressure, *INR* International normalized ratio, *PT* Prothrombin time

Means with standard deviation in parentheses for continuous variables

^a^Absolute numbers with percentages in parentheses for categorical variables

### Statistical analysis

For comparisons of proportions between groups for categorical variables, the Chi-squared Test of Independence and Fisher’s Exact Test were used. For comparisons between groups for continuous variables, the Wilcoxon Rank Sum Test was used. In order to test for significant effect of group on mean biopsy adequacy (number of glomeruli per biopsy procedure), ANCOVA (analysis of variance controlling for other predictors of interest by including them in the model as covariates) was used. The covariates of interest that were included in the multivariable models were age, gender, systolic pressure, International Normalized Ratio (INR), platelet count, glomerular filtration. Rate (GFR), and hemoglobin. Statistical analysis was performed using SAS statistical analysis software for Windows (version 9.4) (SAS Institute, Inc., Cary, NC). Significance level alpha was set a priori at 0.05.

## Results

446 kidney biopsies performed during the time of interest, which included biopsies of 299 native and 147 transplant kidneys. 122 biopsies were performed by nephrology (attendings and fellows) and 324 were performed by interventional radiology (attendings and fellows). All biopsies were performed using real-time ultrasound guidance except for 13 which were performed using CT guided technique. The most frequent complications were microscopic hematuria (6.8%), need for blood transfusion (4.1%), gross hematuria (2.9%). The mean number of glomeruli obtained per biopsy procedure was 26.8.

Descriptive data for native and transplant kidney biopsies, and comparisons between the groups, are summarized in Table 2. Nephrology used 16-gauge needle for 99.17% of their biopsies and IR used an 18-gauge needle for 90.03% of their biopsies, resulting in a significant difference in needle gauge proportions between groups (*p* < 0.0001). There was a significantly greater number of core samples obtained by IR (mean = 3.59, std.dev. = 1.49) compared to nephrology (mean = 2.47, std.dev = 0.79), p < 0.0001. IR used moderate sedation (95.99%) or general anesthesia (1.85%) for the procedures more often than nephrology, which used them 0.82% and 0.82%, respectively, resulting in a statistically significant difference in sedation type proportions between groups (*p* < 0.0001). Trainees (residents or fellows) participated in the biopsy procedures significantly more often in nephrology compared to IR (97.4% versus 69.04%, *p* < 0.0001). No significant difference was found regarding the presence of arteries on the histologic analysis of samples obtained from IR or nephrology.

**Table 2:** Adequacy and complications rates in kidney biopsies performed by interventional radiology and nephrology

	Interventional Radiology (*n* = 324)	Nephrology (*n* = 122)	*p*-value
**Needle Gauge**^a^			** < 0.0001**
16	9 (2.80)	120 (99.17)	
18	289 (90.03)	1 (0.83)	
20	9 (2.80)	0 (0.00)	
Other	14 (4.36)	0 (0.00)	
**Number of core biopsy samples**	3.59 (1.49)	2.47 (0.79)	** < 0.0001**
**Number of Light Glomeruli**	26.87 (15.60)	26.80 (16.62)	0.67
**Presence of Arteries**^a^			
Yes	313 (97.51)	118 (96.72)	0.74
**Microscopic hematuria > 24 hours**^a^			
Yes	25 (7.74)	5 (4.13)	0.18
**Gross hematuria > 24 hours**^a^			
Yes	7 (2.17)	6 (4.96)	0.13
**Need for blood transfusion**^a^			
Yes	12 (3.72)	6 (4.96)	0.59
**Angiography**^a^			
Yes	4 (1.24)	1 (0.83)	1.0
**Nephrectomy**^a^			
Yes	0 (0.00)	0 (0.00)	
**Readmission for bleeding**^a^			
Yes	3 (0.95)	1 (1.06)	1.0
**Urinary tract infection**^a^			
Yes	10 (3.10)	2 (1.65)	0.53
**Biopsy Site Infection**^a^			
Yes	0 (0.00)	0 (0.00)	
**Need for Pain Medications**^a^			
Yes	38 (11.76)	19 (15.70)	0.27
**Pain Lasting More Than 12 Hours**^a^			
Yes	46 (14.24)	19 (15.70)	0.70
**Arteriovenous fistula**^a^			
Yes	0 (0.00)	0 (0.00)	
**Puncture of Liver, Pancreas, Spleen**^a^			
Yes	0 (0.00)	0 (0.00)	

Means with standard deviation in parentheses for continuous variables

^a^Absolute numbers with percentages in parentheses for categorical variables

Overall, for both native and transplant kidneys, patients biopsied by IR more often had a statistically significant higher International Normalized Ratio (INR) (*p* = 0.0002), higher prothrombin time (PT) (*p* < 0.0001), lower glomerular filtration rate (GFR) (*p* = 0.01), higher serum creatinine (*p* = 0.04), lower hemoglobin (*p* < 0.0001), and lower hematocrit (*p* < 0.0001) than those biopsied by nephrology. Despite these findings, there was no significant difference in post biopsy complications, including gross hematuria, microscopic hematuria, need for blood transfusion, need for angiography, need for nephrectomy, readmission for bleeding, urinary tract infection, biopsy site infection, need for pain medications, pain lasting for more than 12 h, creation of arteriovenous fistula, or inadvertent puncture of liver, pancreas, or spleen between IR and nephrology. The biopsy adequacy between the two groups was also similar.

Descriptive data and comparison between nephrology and IR groups for the native kidney biopsy subset only are summarized in Table 3 and Table 4. Regarding biopsy adequacy and complication rates, no significant difference was found between biopsies performed by nephrology or IR. For native biopsies only, there was no significant difference in likelihood of complication between groups, after adjustment for covariates of interest (OR = 1.01, C.I. = (0.42, 2.41), *p* = 0.99). For native biopsies only, there was no significant difference in mean number of glomeruli obtained per biopsy procedure between groups, after adjustment for covariates of interest (F(1,251) = 0.40, *p* = 0.53). No significant difference was found regarding the presence of arteries on the histologic analysis of samples obtained from IR or nephrology.

**Table 3:** Baseline patient characteristic for native kidney biopsy between Interventional Radiology and Nephrology

	Intervention Radiology (*n* = 180)	Nephrology (*n* = 119)	*p*-value
**Age (years)**	57.38 (17.77)	56.16 (19.63)	0.56
**Systolic Blood Pressure (mmHg)**	133.50 (19.97)	129.78 (16.31)	0.11
**Diastolic Blood Pressure (mmHg)**	75.63 (13.65)	72.07 (14.18)	**0.04**
**INR**	1.03 (0.14)	0.99 (0.11)	**0.01**
**PT (sec)**	12.03 (1.64)	11.39 (2.86)	** < 0.0001**
**BUN (mg/dl)**	43.68 (25.15)	39.82 (27.20)	**0.04**
**Platelet Count (x1000µl)**	254.94 (86.92)	247.24 (84.48)	0.62
**GFR (mg/dl)**	35.59 (29.06)	44.84 (33.20)	**0.01**
**Creatinine (ml/min)**	3.19 (3.35)	3.17 (5.70)	0.06
**Hemoglobin (g/dl)**	11.03 (2.25)	11.98 (2.38)	**0.002**
**Hematocrit (%)**	32.94 (6.57)	35.32 (6.75)	**0.01**
**Gender**^a^			0.59
Male	107 (59.44)	67 (56.30)	
Female	73 (40.56)	52 (43.70)	
**Race/Ethnicity**^a^			0.47
White	170 (94.44)	108 (90.76)	
Black	1 (0.56)	1 (0.84)	
Other	9 (5.00)	10 (8.40)	
**Days Prior to Procedure Labs Collected**^a^			**0.002**
Less than or equal to 2 weeks	131 (73.60)	105 (88.24)	
Greater than 2 weeks	47 (26.40)	14 (11.76)	
**Type of Sedation**^a^			** < 0.0001**
No Sedation (Local Only)	0 (0.00)	117 (98.32)	
Moderate Sedation (Versed, fentanyl, etc.)	177 (98.33)	1 (0.84)	
General Anesthesia	3 (1.67)	1 (0.84)	
**Use of Opioids Prior to Procedure**^a^			0.32
No	153 (85.00)	105 (88.98)	
Yes	27 (15.00)	13 (11.02)	
**Fellow Involved in Procedure**^a^			** < 0.0001**
No	58 (32.22)	2 (1.68)	
Yes	122 (67.78)	117 (98.32)	
**Biopsy Performed Using CT Guidance**^a^			**0.004**
No	168 (93.85)	119 (100.00)	
Yes	11 (6.15)	0 (0.00)	
**Kidney**^a^			** < 0.0001**
Right	60 (33.52)	14 (11.76)	
Left	119 (66.48)	105 (88.24)	

Means with standard deviation in parentheses for continuous variables

^a^Absolute numbers with percentages in parentheses for categorical variables

**Table 4:** Adequacy and complications rates in native kidney biopsies performed by interventional radiology and nephrology

	Intervention Radiology (*n* = 180)	Nephrology (*n* = 119)	*p*-value
**Needle Gauge**^a^			** < 0.0001**
16	3 (1.69)	117 (99.15)	
18	159 (89.33)	1 (0.85)	
20	6 (3.37)	0 (0.00)	
Other	10 (5.62)	0 (0.00)	
**Number of core biopsy samples**	3.65 (1.44)	2.46 (0.80)	** < 0.0001**
**Number of Light Glomeruli**	25.87 (16.62)	27.03 (16.71)	0.58
**Presence of Arteries**^a^			
Yes	173 (96.65)	115 (96.64)	1.0
**Microscopic hematuria > 24 hours**^a^			
Yes	14 (7.82)	5 (4.24)	0.22
**Gross hematuria > 24 hours**^a^			
Yes	4 (2.23)	6 (5.08)	0.20
**Need for blood transfusion**^a^			
Yes	7 (3.91)	6 (5.08)	0.63
**Angiography**^a^			
Yes	3 (1.68)	1 (0.85)	1.0
**Nephrectomy**^a^			
Yes	0 (0.00)	0 (0.00)	
**Readmission for bleeding**^a^			
Yes	2 (1.12)	1 (1.10)	1.0
**Urinary tract infection**^a^			
Yes	4 (2.23)	2 (1.69)	1.0
**Biopsy Site Infection**^a^			
Yes	0 (0.00)	0 (0.00)	
**Need For Pain Medications**^a^			
Yes	18 (10.06)	19 (16.10)	0.12
**Pain Lasting More Than 12 Hours**^a^			
Yes	23 (12.85)	19 (16.10)	0.43
**Arteriovenous fistula**^a^			
Yes	0 (0.00)	0 (0.00)	
**Puncture of Liver, Pancreas, Spleen**^a^			
Yes	0 (0.00)	0 (0.00)	

Means with standard deviation in parentheses for continuous variables

^a^Absolute numbers with percentages in parentheses for categorical variables

Subset analysis of transplant kidney biopsy comparisons between nephrology and IR were limited, since only three transplant kidneys were biopsied by nephrology, and 144 were biopsied by IR.

The most common indication for biopsy was elevated creatinine (51.3%), followed by proteinuria (41.7%) and other (6.9%). We did not find any difference in complication rates between these three groups. More specifically, there was no. significant difference in likelihood of complication between elevated creatinine and proteinuria groups (OR = 1.08, C.I. = (0.57, 2.02), *p* = 0.82), between elevated creatinine and “other” groups (OR = 1.15, C.I. = (0.33, 4.06), *p*. = 0.83), or between proteinuria and “other” groups (OR = 1.07, C.I. = (0.30, 3.85), *p* = 0.92). Logistic regression analysis of complication rates is presented in Table 5. Univariate and multivariate logistic regression analysis of complication rates is presented in Table 6.

**Table 5:** Logistic regression analysis of complication rates of native and transplant kidney biopsies performed by interventional radiology and nephrology

Odds Ratio Estimates				
Effect	**Odds Ratio**	**95% Confidence Limits**		***P*-value**
Elevated Creatinine vs Proteinuria	1.076	0.573	2.022	0.8200
Elevated Creatinine vs Other	1.149	0.326	4.056	0.8286
Proteinuria vs Other	1.068	0.296	3.849	0.9196

**Table 6:** Univariate and multivariate logistic regression analysis of complication rates of native kidney biopsies performed by interventional radiology and nephrology

Odds Ratio Estimates				
Effect	**Odds Ratio**	**95% Confidence Limits**		***P*-value**
Univariate:				
Interventional Radiology vs Nephrology	1.049	0.489	2.250	0.9023
Multivariate:				
Interventional Radiology vs Nephrology	1.005	0.420	2.405	0.9913
Female vs Male	0.977	0.400	2.388	0.9597
Age	0.997	0.973	1.021	0.8054
SBP	1.019	0.996	1.042	0.1057
INR	12.941	0.578	289.940	0.1065
Platelet Count	0.998	0.993	1.003	0.3434
GFR	0.995	0.977	1.014	0.6151
Hgb	0.930	0.748	1.157	0.5159

Legend: *SBP* Systolic blood pressure, *INR* International normalized ratio, *GFR* Glomerular filtration rate, *Hgb* Hemoglobin.

## Discussion

The importance of percutaneous kidney biopsy as a diagnostic tool for kidney disease was first highlighted in the landmark publication by Iverson and Brun in 1951 [1]. The technique was refined and popularized by Robert Kark and led to its widespread acceptance [8]. Indications for performing kidney biopsy vary among nephrologists. The overall rates of native kidney biopsy are about 175 per million population in the United States [9, 10]. Historically nephrologists have performed most kidney biopsies and biopsy has been an integral part of the fellowship training of future nephrologists. Over the last two decades for various reasons there has been a steady increase in kidney biopsies performed by interventional radiologists [11]. Our study retrospectively looked at the kidney biopsies performed at our institution in the last six years with an aim to characterize the safety and adequacy of biopsies between those performed by nephrologists versus those by interventional radiologists.

In our study it was striking to see that nephrologists almost exclusively used 16-gauge needle and IR used 18-gauge needle for biopsy. This is similar to trends seen in earlier studies where there has been increasing use of smaller 18-gauge needle by IR [12]. There are more glomeruli obtained per biopsy with use of larger needles (14 or 16-gauge) as compared to 18-gauge needle but is also associated with higher risk of blood transfusion [13, 14]. However in our study we did not notice any difference in biopsy adequacy or rates of complications based on the gauge of needle used, though this was not a priori assumption at the start of our study. We should also point out that the complication rates were very small in our study. We did find that the number of cores obtained by IR was statistically higher than those obtained by nephrology. Patients biopsied by IR also had a higher risk of bleeding (higher INR, higher prothrombin time, higher serum creatinine, lower hemoglobin (*p* < 0.0001) but there was no difference in the two groups when looked at biopsy adequacy or complication rates. Interventional radiologist at our institution used small size needle (18-gauge) as compared to nephrology who exclusively used 16-gauge needle, and this may account for similar rates of complication as theoretically a smaller needle size may reduce the complication.

Another interesting finding was that when IR fellows were involved in the biopsy, a fewer number of glomeruli were obtained as compared to when the biopsy was done by IR attending, but the risk of complications and overall adequacy of biopsy sample was not affected. Number of biopsies when nephrology fellows were not involved were too low to make any conclusive comment for this subset.

Sparse data exists comparing kidney biopsy outcomes between nephrology fellows and those done by radiology, with one small study showing equal efficacy and another larger study showing better success of kidney biopsy performed by IR as compared to nephrologist or surgeons [7, 15]. Another study which compared ultrasound marked blind biopsy with real time ultrasound guided biopsies by nephrologists and radiologists did not show any difference among the three groups [16]. There is ongoing debate within nephrology training programs on the continued utility of requirements to train nephrology fellows in skills of percutaneous kidney biopsy. Our finding that biopsies performed by nephrology (nephrology fellows under supervision by nephrology attending) or IR are similar for its diagnostic yield and complication rates can be interpreted in different ways. Interest in nephrology as a subspecialty has been waning and few argue that the procedural aspect of nephrology may attract future trainees and that there is need to continue to train fellows in kidney biopsies [17]. Ability to perform kidney biopsy is a core skill set which may be very useful for a nephrologist in rural setting with limited resources [18]. Our study gives credence to that argument as biopsy outcomes by nephrology fellows are similar to those performed by IR suggesting ability to master a skill with adequate training and supervision. On the other hand, in one survey it was found that half of graduating nephrology fellows perform ten or less native and transplant kidney biopsies and few do not perform biopsy after graduation at all [19, 20]. These trends may be driven by various factors like time constraints due to high clinical volumes, time consuming procedure, heavy burden of documentation and low reimbursements [21]. Without continual experience one can argue that competence declines and it becomes a patient safety issue, especially when there are other services (like IR) that perform renal biopsy with similar diagnostic yield as shown in our study.

Several limitations should be kept in mind while drawing conclusions from our analysis. This was a single center, retrospective study and not a randomized trial. There is also a difference in time period when nephrologist performed the majority of biopsies (2015 to 2017) compared to the current time when all biopsies are exclusively performed by IR (2018 to 2021). However, we do not feel that this should affect our results as the technique has not changed. We do not have data on the exact number of passes made which can be an important factor for complications, as studies have shown increased rate of complications with greater than five passes [22]. However, we did not experience any immediate hematoma documented by ultrasound. We also did not have a mechanism to look into the cost effectiveness, punctuality time from requisition to acquisition, and patient satisfaction between biopsies performed by nephrology and IR. Another limitation is that, at our institution we do not routinely perform ultrasound or hemoglobin check in clinically asymptomatic patients post-biopsy and we could have missed some complications like asymptomatic hematoma or asymptomatic arteriovenous fistula (AVF). Prior studies have shown about a 14% rate of AVF formation after kidney biopsies [23]. Post-biopsy AVF are usually clinically silent and resolve spontaneously (70% cause no symptoms and resolve spontaneously within weeks) [24].

## Conclusion

Our results suggest that there is no significant difference in the adequacy or complication rates between kidney biopsies performed by IR or nephrology. This conclusion may indicate that kidney biopsies can be performed safely with adequate results either by IR or nephrologists depending on each institution’s resources and expertise.

## Data Availability

The data that support the findings of this study are available on reasonable request from the corresponding author. The data are not publicly available due to privacy or ethical restrictions.
